# Sport-specific injury profiles and associated factors in elite wrestlers and judokas

**DOI:** 10.3389/fpubh.2026.1910441

**Published:** 2026-08-28

**Authors:** Yunus Sadır, Faik Öz, Suzan Dal, Selin Biçer Baikoğlu, İrfan Kara, İzzet Karakulak, Hakan Bilgen, Yusuf Kurt, Cüneyt Şensoy, Osman Imamoğlu, Serdar Koç

**Affiliations:** 1 Ministry of National Education of the Republic of Türkiye, Erzurum Provincial Directorate of National Education, Pasinler District Directorate of National Education, Pasinler Atatürk Boarding Secondary School, Erzurum, Türkiye; 2 Faculty of Sport Sciences, Avrasya University, Trabzon, Türkiye; 3 Department of Exercise and Sport Sciences for Individuals With Disabilities, Faculty of Sports Sciences, Istanbul University-Cerrahpaşa, Istanbul, Türkiye; 4 Division of Sports Health Sciences, Department of Coaching Education, Faculty of Sports Sciences, Istanbul University-Cerrahpaşa, Istanbul, Türkiye; 5 Department of Sports Management, Faculty of Sports Sciences, Istanbul Gelişim University, Istanbul, Türkiye; 6 Department of Sports Management, Faculty of Sports Sciences, Mardin Artuklu University, Mardin, Türkiye; 7 Faculty of Sports Sciences, Lokman Hekim University, Ankara, Türkiye; 8 Faculty of Sports Sciences, Inönü University, Malatya, Türkiye; 9 Graduate School of Health Sciences, Gazi University, Ankara, Türkiye; 10 Department of Sports Management, Yaşar Dogu Faculty of Sports Sciences, Ondokuz Mayis University, Samsun, Türkiye; 11 Republic of Türkiye Ministry of National Education, Muş Provincial Directorate of National Education, Muş, Türkiye

**Keywords:** athletic exposure, combat sports, injury epidemiology, injury-associated factors, multiple injury episodes, musculoskeletal injuries, weekly training frequency

## Abstract

**Background:**

Wrestling and judo involve repeated physical contact and substantial musculoskeletal demands. Although the two sports share structural similarities, differences in sport-specific demands may be associated with distinct injury characteristics. This study compared self-reported injury profiles between elite wrestlers and judokas and examined factors associated with reporting multiple injury episodes.

**Methods:**

This cross-sectional comparative study included 289 Turkish National Team athletes, comprising 143 wrestlers and 146 judokas. Injury and health-problem data were collected retrospectively across the athletes' competitive careers using a structured self-report form. Athlete-level comparisons, ordinal logistic regression, generalized estimating equation models accounting for within-athlete clustering, false discovery rate correction, and multivariable binary logistic regression were applied.

**Results:**

Wrestlers reported a significantly higher weekly training frequency than judokas (*p* <.001). Longer athletic experience was associated with greater odds of classification in a higher self-reported injury-episode category in both sports. After accounting for within-athlete clustering and false discovery rate correction, judokas had lower odds of reporting neck injuries [OR = 0.27, 95% CI (0.12, 0.58), adjusted *p* =.016] and other shoulder injuries [OR = 0.43, 95% CI (0.24, 0.77), adjusted *p* =.049] than wrestlers. No statistically significant sport effect, injury-setting effect, or sport × setting interaction was detected for the reported injury and health-problem categories. Sprain, muscle strain, and skin infection were the most frequently reported categories descriptively. At the athlete level, athletic experience [OR = 1.20, 95% CI (1.08, 1.34), *p* <.001] and weekly training frequency [OR = 1.14, 95% CI (1.04, 1.26), *p* =.006] were positively associated with reporting multiple injury episodes. Judokas had lower odds of reporting multiple injury episodes than wrestlers [OR = 0.70, 95% CI (0.53, 0.93), *p* =.017].

**Conclusion:**

Limited sport-related differences were identified in self-reported injury-location patterns, with significant differences remaining only for neck injuries and other shoulder injuries after correction for multiple comparisons. Athletic experience and weekly training frequency were associated with reporting multiple injury episodes. The findings should be interpreted as retrospective self-reported injury frequencies and associations rather than exposure-adjusted injury incidence rates or causal relationships.

## 1 Introduction

Wrestling and judo are high-intensity combat sports characterized by frequent physical contact and a substantial risk of musculoskeletal injury (1–3). Elite judo also requires high levels of aerobic and anaerobic fitness, strength, and power to meet the physiological demands of training and competition (4). Understanding injury mechanisms is critical for effective prevention; however, the technical complexity and variability of combat sports make injury patterns difficult to interpret (5). Injuries in combat sports may be associated with repetitive physical contact, high-impact collisions, inadequate preparation, and lapses in concentration during training and competition. Despite advances in prevention strategies, injury risk remains high, potentially reflecting the aggressive and high-intensity nature of these sports, as well as additional physiological stressors such as rapid weight loss and dehydration (6). These disciplines involve rapid and repetitive offensive and defensive actions that impose substantial mechanical stress on both upper and lower body structures, potentially contributing to cumulative tissue fatigue and injury susceptibility (7, 8). In combat sports, injuries commonly involve musculoskeletal and soft tissue damage requiring medical attention during training or competition. Although wrestling is associated with a high incidence of orthopedic injuries, reported injury patterns vary considerably because of differences in study design, injury definitions, surveillance methods, athlete populations, and combat sport modalities (9–11). Injury rates in wrestling and judo may also differ according to competitive level, exposure characteristics, and assessment context. Although injury patterns may appear similar, important differences emerge between training and competition contexts as well as between wrestling and judo. Therefore, the present study aimed to compare retrospectively reported injury characteristics across the competitive careers of elite wrestlers and judokas and to identify factors associated with reporting multiple injury episodes (12).

Although previous studies have reported injury patterns in wrestling and judo, most investigations have examined these sports separately and primarily focused on general injury frequency or specific injury regions. Comparative evidence regarding how injury location, injury type, repeated injury episodes, and training-related factors differ between elite wrestlers and judokas remains limited. In particular, relatively little attention has been given to the combined evaluation of injury characteristics and factors associated with injury occurrence within elite combat sport athletes. Because wrestling and judo involve different technical structures, movement patterns, and biomechanical demands, injury profiles may differ considerably between these disciplines. Understanding these sport-specific differences may help improve injury prevention approaches and training practices in elite combat sports. Therefore, the present study aimed to compare injury characteristics between elite wrestlers and judokas and to examine factors associated with multiple self-reported injury episodes.

## 2 Materials and Methods

### 2.1 Participants

A total of 289 elite combat sport athletes, including 143 wrestlers and 146 judokas, participated in this cross-sectional comparative study. All athletes were active members of the Turkish National Teams competing in youth and senior categories during the data collection period. Participants were recruited using purposive sampling. Inclusion criteria required athletes to hold an active national athlete license, participate regularly in organized training and official competitions, and maintain active competitive status throughout the study period. Athletes who were unavailable due to long-term injury or rehabilitation were excluded from participation. The selection of national-level athletes was intended to provide injury data representative of elite combat sport participation and high-performance training exposure.

### 2.2 Study design

This study employed a cross-sectional comparative design to examine sport-specific injury characteristics among elite wrestlers and judokas. Injury profiles were compared between the two combat sport disciplines according to injury location, injury and health-problem category, number of reported injury episodes, and injury context (training vs. competition). In addition, ordinal logistic regression analysis was performed to evaluate the association between athletic experience and reported injury episode frequency, while multivariate logistic regression analysis was used to examine training-related and sport-specific factors associated with reporting multiple injury episodes.

### 2.3 Data collection procedure

A structured data collection form developed by the researchers was used to obtain demographic, training-related, and injury-related information from the participants. Prior to implementation, the form was reviewed by experts in sports medicine and combat sports coaching to improve content clarity and relevance. The data collection instrument consisted of two sections: a personal information form including demographic and training-related variables, and an injury follow-up form designed to assess injury characteristics. Injury and health-problem categories were predefined in the form, and athletes selected the category that they believed best represented each reported injury or health problem. The form was completed under researcher supervision. The use of predefined categories, expert review, and researcher supervision was intended to improve the clarity of the categories and reduce potential classification errors. However, the reported injuries were not independently verified through medical records, clinical examination, imaging findings, or physician diagnosis, and no clinical reclassification was performed. Therefore, although measures were taken to improve category clarity, the possibility of misclassification bias could not be eliminated. Athletes retrospectively reported injuries and health problems experienced throughout their competitive sports careers, including injury or health-problem category, anatomical location, number of reported injury episodes, and injury context (training vs. competition).

### 2.4 Measures

Study variables were categorized as demographic, training-related, and injury-related measures. Demographic and anthropometric variables included age (years), height (cm), body weight (kg), and body mass index (BMI; kg/m^2^). Training-related variables included athletic experience (years) and weekly training frequency (days/week). Weekly training frequency was defined as the number of training days per week and was considered a limited proxy for training exposure rather than a comprehensive measure of training load. Training duration, training intensity, session volume, competition exposure, athlete-exposure hours, session-RPE, and recovery status were not assessed. Therefore, exposure-adjusted injury incidence rates could not be calculated, and the findings should be interpreted as retrospective self-reported injury frequencies rather than estimates of injury risk or incidence. Injury-related variables included injury occurrence, number of reported injury episodes, injury and health-problem category, anatomical location, and injury context. Injury occurrence was defined as reporting at least one musculoskeletal or soft-tissue injury requiring medical attention and/or resulting in modified sports participation. The number of reported injury episodes was categorized as one episode, two episodes, and three or more episodes for ordinal regression analyses. All injury-related information referred to events experienced throughout the athletes' competitive sports careers and was collected retrospectively through self-report.

Reported injury and health-problem categories included sprain, muscle strain, contusion, fracture, dislocation/subluxation, ligament injury, cartilage injury, skin infection, and other minor conditions. Skin infection was treated as a combat-sport-related health problem rather than a musculoskeletal injury. Information regarding injury mechanism, acute versus overuse onset, and time-loss severity was not systematically collected; therefore, these dimensions could not be included in the classification. Anatomical locations were categorized into major body regions, including neck/back/waist, shoulder region, head/face, upper extremity, lower extremity, trunk, and skin-related conditions. Injury and health-problem categories and anatomical locations were predefined in the data collection form. Because the information was based on retrospective athlete self-report and was not clinically verified, the reported classifications may be subject to recall and misclassification bias. Because athletes could report more than one event during their competitive sports careers, the variables were recorded at both the athlete level and the episode level. Athlete-level variables included injury occurrence, number of reported episodes, and demographic and training-related characteristics, whereas episode-level variables included anatomical location, injury or health-problem category, and context.

### 2.5 Statistical analysis

All statistical analyses were performed using IBM SPSS Statistics, version 27.0. Continuous variables were summarized using means and standard deviations, whereas categorical variables were summarized using frequencies and percentages. Before conducting parametric analyses, distributional assumptions were assessed using skewness and kurtosis values together with visual inspection of the data distributions. Independent-samples *t*-tests were used to compare demographic, anthropometric, and training-related characteristics between wrestlers and judokas. Effect sizes for these group comparisons were calculated using Cohen's d.

To account for the clustering of multiple injury episodes within the same athlete, cluster-adjusted regression models were used for episode-level analyses. Associations between sport type and each reported injury location were examined using generalized estimating equation models with a binary distribution and logit link function. Athlete identification number was specified as the clustering variable, and odds ratios with 95% confidence intervals were calculated. Because multiple injury-location comparisons were performed, *p*-values were adjusted using the false discovery rate procedure. Cluster-adjusted binary logistic regression models were used to examine the associations of sport type, injury setting, and the sport type × injury setting interaction with each reported injury and health-problem category.

Ordinal logistic regression analysis was used to examine the association between athletic experience categories and the number of self-reported injury episodes among wrestlers and judokas. The ordinal outcome was categorized as one injury episode, two injury episodes, and three or more injury episodes.

A multivariable binary logistic regression analysis was conducted at the athlete level to examine factors associated with reporting multiple injury episodes. Multiple injury episode status was coded as 0 = one reported injury episode and 1 = two or more reported injury episodes. This classification reflects the cumulative number of self-reported injury episodes during the athlete's competitive career and does not indicate recurrence of the same injury, anatomical region, or diagnosis. Age, athletic experience, weekly training frequency, body mass index, and sport type were entered simultaneously into the model using the enter method.

Multicollinearity among the independent variables was assessed using tolerance and variance inflation factor values. Although age and athletic experience were conceptually related, both variables were retained in the model because the multicollinearity diagnostics indicated no problematic overlap and each variable represented a distinct athlete characteristic. Odds ratios with 95% confidence intervals were calculated for the regression analyses. Model fit was evaluated using the Hosmer-Lemeshow goodness-of-fit test, and Nagelkerke R^2^ was reported as a pseudo-R^2^ measure. Statistical significance was set at *p* <.05.

## 3 Results

The results of the study are presented according to demographic characteristics, injury distributions, and factors associated with reporting multiple injury episodes among wrestling and judo athletes.

Table 1 presents the comparison of demographic, anthropometric, and training-related characteristics between wrestling and judo athletes. No significant differences were observed between the groups in terms of age, athletic experience, height, body weight, or body mass index (*p* >.05). However, wrestlers demonstrated significantly higher weekly training frequency compared with judo athletes (*p* <.001, Cohen's d = 0.44), indicating a small-to-moderate practical difference between the groups.

**Table 1:** Comparison of demographic, anthropometric, and training-related characteristics between wrestling and judo athletes.

Variable	Type of sport	Mean	SD	*t*	*p*	Cohen's d
Age (years)	Wrestling	21.80	2.31	−0.36	0.717	−0.04
	Judo	21.90	2.38			
Athletic experience (years)	Wrestling	9.13	1.26	−0.92	0.359	−0.11
	Judo	9.27	1.33			
Weekly training frequency (days/week)	Wrestling	6.65	2.31	3.71	<.001	0.44
	Judo	5.64	2.32			
Height (cm)	Wrestling	173.11	7.31	−0.02	0.982	0.00
	Judo	173.13	7.50			
Body weight (kg)	Wrestling	71.63	10.44	−0.20	0.845	−0.02
	Judo	71.87	10.41			
Body mass index (kg/m^2^)	Wrestling	23.98	2.12	0.04	0.968	0.00
	Judo	23.97	2.15			

Table 2 presents the distribution of athletes according to the number of reported injury episodes across athletic experience categories in wrestling and judo. Each athlete was classified into a single injury-episode category based on the total number of injuries reported throughout his or her competitive career; therefore, the frequencies represent individual athletes rather than injury episodes. In both sport disciplines, athletes with ≥11 years of athletic experience were more frequently represented in the two-episode and three-or-more-episode categories. Specifically, 61.76% of wrestlers and 63.33% of judokas reporting three or more injury episodes had ≥11 years of athletic experience. In contrast, athletes with ≤ 8 years of experience were more commonly represented in the one-episode category. Overall, the descriptive distribution indicates that a greater number of reported injury episodes tended to be observed among athletes with longer competitive experience, which may reflect greater cumulative exposure over prolonged athletic careers.

**Table 2:** Distribution of reported injury episodes according to athletic experience categories in wrestling and judo athletes.

Number of reported injury episodes	Wrestling				Judo			
	≤ 8 years	9–10 years	≥11 years	Total	≤ 8 years	9–10 years	≥11 years	Total
One episode	20	17	16	53	17	24	19	60
% within injury category	37.74	32.08	30.19	100	28.33	40.00	31.67	100
Two episodes	9	22	25	56	8	24	24	56
% within injury category	16.07	39.29	44.64	100	14.29	42.86	42.86	100
Three or more episodes	3	10	21	34	2	9	19	30
% within injury category	8.82	29.41	61.76	100	6.67	30.00	63.33	100
Total athletes	32	49	62	143	27	57	62	146

Values represent individual athletes. Each athlete was classified into one injury-episode category according to the total number of episodes reported during his or her competitive career and contributed to only one cell in the table. All participants reported at least one episode. Percentages were calculated within each injury-episode category. Across all participants, wrestlers reported 454 injury and health-problem episodes, whereas judokas reported 472.

The proportional odds assumption was not violated for either sport, as shown in Table 3. The ordinal logistic regression analysis demonstrated a significant association between years of sports participation and the number of self-reported injury episodes in both wrestlers and judokas. Among wrestlers, athletes with 9–10 years of experience had approximately twice the odds of being classified in a higher injury-episode category compared with athletes with 8 years or less experience [OR = 2.03, 95% CI (1.32, 3.13), *p* =.001]. Wrestlers with 11 years or more experience had 3.60 times greater odds of being classified in a higher reported injury-episode category [95% CI (2.40, 5.39), *p* <.001]. Among judokas, athletes with 9–10 years of experience had 1.94 times greater odds of being classified in a higher injury-episode category than athletes with 8 years or less experience [95% CI (1.23, 3.04), *p* =.003]. Judokas with 11 years or more experience had 3.13 times greater odds of being classified in a higher injury-episode category [95% CI (2.05, 4.79), *p* <.001].

**Table 3:** Test of parallel lines for ordinal logistic regression models.

Sport Type	-2 Log Likelihood Null Hypothesis	-2 Log Likelihood General Model	Chi-Square	df	*p*
Wrestling	412.84	409.76	3.08	2	0.214
Judo	398.17	395.94	2.23	2	0.328

The proportional odds assumption was evaluated using the Test of Parallel Lines. Non-significant results (p >.05) indicated that the assumption was not violated for either model, supporting the use of ordinal logistic regression.

Table 4 presents cluster-adjusted associations between sport type and reported injury locations. After accounting for the clustering of multiple injury episodes within athletes and applying false discovery rate correction, judo athletes had significantly lower odds of reporting neck injuries than wrestlers [OR = 0.27, 95% CI (0.12, 0.58), FDR-adjusted *p* =.016]. Judo athletes also had significantly lower odds of reporting other shoulder injuries than wrestlers [OR = 0.43, 95% CI (0.24, 0.77), FDR-adjusted *p* =.049]. No other injury-location differences remained statistically significant after false discovery rate correction.

**Table 4:** Cluster-adjusted associations between sport type and reported injury locations.

Injury area	Injury location	Wrestling, *n* (%)	Judo, *n* (%)	OR	95% CI	GEE *p*	FDR–adjusted *p*
Neck/Back/Waist	Neck	26 (5.7)	8 (1.7)	0.27	0.12–0.58	<.001	0.016^*^
	Back	12 (2.6)	23 (4.9)	1.96	1.00–3.85	0.051	0.163
	Waist	31 (6.8)	34 (7.2)	1.06	0.64–1.75	0.824	0.969
Shoulder area	Shoulder and collarbone	16 (3.5)	26 (5.5)	1.53	0.83–2.83	0.176	0.411
	Rotator cuff	14 (3.1)	24 (5.1)	1.69	0.86–3.31	0.125	0.329
	Other shoulder injuries	34 (7.5)	16 (3.4)	0.43	0.24–0.77	0.005	0.049^*^
	Dislocation/subluxation	14 (3.1)	17 (3.6)	1.19	0.60–2.35	0.613	0.969
Head/Face	Eyes	15 (3.3)	23 (4.9)	1.50	0.78–2.87	0.222	0.467
	Nasal region	10 (2.2)	22 (4.7)	2.21	0.99–4.90	0.052	0.163
	Ears	19 (4.2)	21 (4.4)	1.04	0.55–1.99	0.901	0.969
Arm/Elbow/Wrist/Fingers	Fingers	18 (4.0)	19 (4.0)	1.05	0.55–1.98	0.891	0.969
	Elbow	13 (2.9)	31 (6.6)	2.39	1.25–4.55	0.008	0.056
	Wrist	19 (4.2)	20 (4.2)	1.02	0.55–1.89	0.958	0.969
	Arm muscles	19 (4.2)	17 (3.6)	0.85	0.44–1.66	0.643	0.969
Knee/Ankle/Toes	Knee region	47 (10.4)	32 (6.8)	0.63	0.39–1.01	0.054	0.163
	Ankle	49 (10.8)	30 (6.4)	0.56	0.35–0.89	0.013	0.070
	Big toe	16 (3.5)	16 (3.4)	0.95	0.49–1.84	0.887	0.969
Trunk	Chest	21 (4.6)	25 (5.3)	1.17	0.64–2.16	0.609	0.969
	Abdominal region	22 (4.8)	25 (5.3)	1.10	0.61–1.97	0.756	0.969
Skin	Abrasion	18 (4.0)	21 (4.4)	1.13	0.61–2.12	0.696	0.969
	Inflammation	21 (4.6)	22 (4.7)	1.01	0.56–1.84	0.969	0.969

^*^ indicates statistical significance after false discovery rate correction (FDR-adjusted *p* <.05).

Table 5 presents cluster-adjusted associations of sport type and injury setting with reported injury and health-problem categories. After accounting for the clustering of multiple injury episodes within athletes, no significant sport effect, injury-setting effect, or sport type × injury setting interaction was observed for any category (*p* >.05). Sprain, muscle strain, and skin infection remained the most frequently reported categories descriptively in both sport disciplines.

**Table 5:** Cluster–adjusted associations of sport type and injury setting with reported injury and health-problem categories.

Injury and health–problem category	Wrestling training, *n* (%)	Wrestling competition, *n* (%)	Judo training, *n* (%)	Judo competition, *n* (%)	Sport OR	95% CI	Sport *p*	Context OR	95% CI	Context *p*	Sport × context *p*
Sprain	56 (20.7)	28 (15.3)	72 (23.7)	33 (19.6)	1.17	0.80–1.72	0.410	0.69	0.41–1.17	0.165	0.659
Muscle strain	44 (16.2)	24 (13.1)	51 (16.8)	32 (19.0)	1.05	0.70–1.59	0.806	0.78	0.49–1.25	0.303	0.218
Skin infection	36 (13.3)	24 (13.1)	46 (15.1)	20 (11.9)	1.16	0.72–1.87	0.530	0.99	0.56–1.75	0.981	0.513
Contusion	30 (11.1)	21 (11.5)	48 (15.8)	21 (12.5)	1.52	0.93–2.48	0.098	1.04	0.61–1.76	0.885	0.426
Cartilage tear	24 (8.9)	18 (9.8)	32 (10.5)	12 (7.1)	1.21	0.70–2.11	0.498	1.12	0.57–2.21	0.735	0.258
Fracture	22 (8.1)	20 (10.9)	14 (4.6)	13 (7.7)	0.55	0.27–1.10	0.089	1.39	0.75–2.56	0.295	0.661
Dislocation/subluxation	20 (7.4)	16 (8.7)	15 (4.9)	14 (8.3)	0.65	0.33–1.29	0.220	1.20	0.62–2.33	0.589	0.495
Ligament tear (knee)	19 (7.0)	17 (9.3)	12 (3.9)	11 (6.5)	0.56	0.26–1.18	0.125	1.39	0.69–2.81	0.352	0.752
Other injuries	20 (7.4)	15 (8.2)	14 (4.6)	12 (7.1)	0.63	0.30–1.30	0.212	1.07	0.48–2.36	0.874	0.487
Total	271 (100)	183 (100)	304 (100)	168 (100)							

Values are presented as *n* (%), with percentages calculated within each sport and injury–setting group. Odds ratios (ORs), 95% confidence intervals (CIs), and *p* values were estimated using cluster–adjusted binary logistic regression models accounting for multiple injury episodes reported by the same athlete. Wrestling was used as the reference category for sport type, and training was used as the reference category for injury setting. The sport × context *p* value represents the interaction between sport type and injury setting. Statistical significance was set at *p* <.05.

Prior to conducting the multivariate logistic regression analysis, multicollinearity diagnostics were performed. As shown in Table 6, all tolerance values exceeded 0.20 and all VIF values were below 5.00, indicating no evidence of problematic multicollinearity among the independent variables.

**Table 6:** Multicollinearity diagnostics for independent variables.

Variable	Tolerance	VIF
Age	0.62	1.61
Athletic experience	0.58	1.72
Weekly training frequency	0.84	1.19
Body Mass Index	0.91	1.10
Sport type	0.88	1.14

All tolerance values exceeded 0.20 and all VIF values were below 5.00, indicating no evidence of problematic multicollinearity among the independent variables included in the logistic regression model.

Table 7 presents the multivariable binary logistic regression analysis of factors associated with reporting multiple injury episodes among elite combat sport athletes. Athletic experience and weekly training frequency were significantly associated with multiple injury episode status (*p* <.01). In addition, judokas had lower odds of reporting multiple injury episodes than wrestlers (OR = 0.70, *p* =.017). Age and body mass index were not significantly associated with multiple injury episode status (*p* >.05). The Hosmer-Lemeshow test indicated no evidence of poor model fit (*p* =.48), and the Nagelkerke R^2^ value was.21.

**Table 7:** Multivariable binary logistic regression analysis of factors associated with reporting multiple injury episodes among elite combat sport athletes.

Variable	B	SE	Wald	*p*	OR (Exp(B))	95% CI for OR
Age	0.042	0.031	1.83	0.176	1.04	[0.98, 1.11]
Athletic experience (years)	0.185	0.052	12.64	<.001	1.20	[1.08, 1.34]
Weekly training frequency (days/week)	0.128	0.047	7.42	0.006	1.14	[1.04, 1.26]
Body Mass Index	0.021	0.058	0.13	0.718	1.02	[0.91, 1.14]
Sport type (Judo = 1, Wrestling = 0)	−0.356	0.149	5.71	0.017	0.70	[0.53, 0.93]
Constant	−2.145	0.842	6.49	0.011	—	—

Model fit statistics: −2 Log Likelihood = 412.36, Nagelkerke R^2^ =.21, and Hosmer–Lemeshow *p* =.48. Multiple injury-episode status was defined as reporting two or more injury episodes during the athlete's competitive sports career. This classification does not indicate recurrence of the same injury, anatomical region, or diagnosis. Of the 289 athletes included in the model, 113 reported one injury episode and 176 reported two or more injury episodes.

## 4 Discussion

The similarity of demographic and anthropometric characteristics between wrestling and judo athletes strengthens the comparability of the injury findings between the two groups. Since variables such as age, body size, and body mass index were largely comparable, the observed differences in injury patterns may reflect sport-specific technical and biomechanical demands rather than demographic variation. However, wrestlers demonstrated higher weekly training frequency, which should be considered when interpreting the observed differences in injury patterns between the groups.

The findings presented in Table 2 support the cumulative exposure perspective, indicating that athletes with longer competitive careers reported a greater cumulative number of injury episodes. This association may partly reflect their longer period of exposure to combat sport participation. Similarly, the ordinal logistic regression findings demonstrated that athletes with longer athletic experience had significantly greater odds of being classified in a higher self-reported injury-episode category in both wrestling and judo. These findings are consistent with contemporary overuse and tissue-fatigue models proposing that repeated submaximal loading may progressively reduce tissue tolerance and increase injury susceptibility over time (7, 13). Combat sport athletes are repeatedly exposed to high-intensity throws, falls, joint-loading actions, and direct physical contact, all of which may contribute to both acute and overuse injury mechanisms. In addition, repetitive loading combined with insufficient recovery may promote cumulative microdamage within muscle-tendon-bone structures, which may contribute to a greater number of injury episodes over prolonged athletic careers (8). The present findings also align with previous evidence indicating that prior injury is strongly associated with subsequent injury occurrence, particularly when residual deficits, altered movement patterns, or incomplete recovery persist following the initial injury (14). Similarly, Hägglund et al. (15) emphasized that injury recurrence remains common among elite athletes exposed to repeated high-intensity training and competition demands across prolonged competitive seasons.

The ordinal logistic regression findings presented in Table 8 further supported this interpretation by demonstrating progressively greater odds of being classified in a higher self-reported injury-episode category across athletic experience categories in both wrestlers and judokas. In particular, athletes with ≥11 years of participation had markedly greater odds of being classified in a higher injury-episode category than athletes with ≤ 8 years of experience, suggesting an association between prolonged combat sport participation and a greater cumulative number of self-reported injury episodes. These findings may primarily reflect the longer cumulative exposure period of athletes with extended competitive careers rather than a greater susceptibility to recurrence of the same injury.

**Table 8:** Ordinal logistic regression analysis of the number of self-reported injury episodes according to athletic experience categories.

Predictor variable	B	SE	Wald χ^2^	OR	95% CI	*p*
Wrestling						
9–10 years	0.71	0.22	10.41	2.03	1.32–3.13	0.001^*^
≥11 years	1.28	0.20	40.52	3.60	2.40–5.39	<.001^**^
Judo						
9–10 years	0.66	0.23	8.54	1.94	1.23–3.04	0.003^*^
≥11 years	1.14	0.21	28.76	3.13	2.05–4.79	<.001^**^

^*^p <.05, ^**^p <.001.

Reference category: ≤ 8 years of experience. Dependent variable: Number of self-reported injury episodes (1 = one episode, 2 = two episodes, 3 = three or more episodes).

The cluster-adjusted findings presented in Table 4 showed that sport-related differences remained statistically significant for neck injuries and other shoulder injuries after false discovery rate correction. Judo athletes had lower odds of reporting neck injuries and other shoulder injuries than wrestlers. The higher reporting frequency of neck injuries among wrestlers may be associated with sustained grappling exchanges, repetitive takedown attempts, defensive resistance maneuvers, and cervical loading during ground-control situations. Previous epidemiological evidence has similarly identified the cervical and spinal regions as important injury sites in wrestling and has shown that injury profiles vary across Olympic combat sports according to their technical and biomechanical demands (1, 11, 16). Shoulder and elbow injuries have also been identified as relevant components of the injury profile in wrestlers (17). Evidence from judo has also demonstrated that injury patterns may vary according to throwing actions, landing impacts, and competition dynamics, although such findings do not directly explain the lower odds observed for other shoulder injuries in the present sample (18, 19). Although numerical differences were observed for elbow, ankle, and knee injuries, these associations did not remain statistically significant after correction for multiple comparisons and should therefore be interpreted cautiously. The findings suggest that sport-related differences may be confined to specific anatomical locations rather than representing a consistent pattern across all injury regions.

Table 5 presents the cluster-adjusted associations of sport type and injury setting with reported injury and health-problem categories. After accounting for the clustering of multiple injury episodes within the same athlete, no statistically significant sport effect was observed for any injury or health-problem category (*p* >.05). Contusions tended to be more frequently reported among judokas than wrestlers [OR = 1.52, 95% CI (0.93, 2.48), *p* =.098], whereas fractures tended to be less frequent among judokas [OR = 0.55, 95% CI (0.27, 1.10), *p* =.089]; however, these associations did not reach statistical significance. Injury setting was also not significantly associated with any of the examined injury and health-problem categories (*p* >.05). Similarly, no significant sport-by-setting interaction effects were observed, indicating that the relationship between training and competition settings and the reported injury and health-problem categories did not differ significantly between wrestlers and judokas.

The findings presented in Table 5 showed no statistically significant effects of sport type, injury setting, or sport type × injury setting interaction on any reported injury or health-problem category. Although numerical differences were observed between wrestlers and judokas, none of the sport effects reached statistical significance after accounting for within-athlete clustering. Wrestlers showed numerically higher frequencies of fractures, ligament injuries, and dislocations, whereas judokas showed numerically higher frequencies of sprains, muscle strains, and contusions. However, these differences should be interpreted descriptively and should not be considered evidence of sport-specific injury and health-problem patterns. Similarly, no statistically significant differences were detected between training and competition settings. Because detailed exposure duration, training intensity, and competition load were not assessed, the findings represent self-reported injury frequencies rather than exposure-adjusted injury incidence rates.

The findings presented in Table 7 showed that athletic experience and weekly training frequency were significantly associated with multiple injury-episode status among elite combat sport athletes. Greater athletic experience was associated with higher odds of reporting two or more injury episodes during the competitive career. Given the cross-sectional and retrospective design, these findings should be interpreted as associations rather than evidence of causal relationships. Longer participation may correspond to a longer period during which injuries could be accumulated and reported. Previous epidemiological evidence has similarly emphasized the role of prolonged exposure in shaping cumulative injury burden in combat sport athletes (20). Weekly training frequency was also positively associated with multiple injury-episode status. This finding is broadly consistent with evidence linking greater training exposure to injury occurrence across athletic populations (21) and with reports highlighting the importance of structured periodisation in combat sport athletes (22). However, weekly training frequency in the present study represented only the number of training days per week and did not capture training duration, intensity, volume, contact exposure, or recovery. Judokas had lower odds of reporting multiple injury episodes than wrestlers after adjustment for the variables included in the model. These findings support cautious interpretation of sport-related differences and highlight the value of monitoring cumulative injury history and weekly training frequency in elite combat sport athletes.

From a practical perspective, coaches, sports medicine practitioners, physiotherapists, and athletic trainers should consider regular monitoring of injury history, weekly training frequency, and cumulative exposure in elite combat sport athletes. For wrestlers, particular attention may be directed toward cervical loading, neck strength, neuromuscular control, and technical execution during takedown and ground-control situations. Shoulder stability, joint mobility, and progressive load management may also be relevant across both sports. Although knee and ankle injuries in wrestlers and shoulder and elbow injuries in judokas may warrant targeted monitoring in practice, the present between-sport differences for these regions did not remain statistically significant after correction for multiple comparisons. Therefore, preventive strategies should be informed by prospective injury surveillance, individualized screening, and sport-specific movement demands rather than descriptive frequencies alone.

## 5 Limitations

Some limitations of the present study should be acknowledged. First, the retrospective and self-reported nature of the injury data may have introduced recall bias and potential underreporting, particularly for less severe injuries experienced earlier in the athletes' careers. Furthermore, injury history was assessed across the athletes' competitive careers rather than within a predefined observation period, which may have increased recall bias and affected the accuracy of self-reported injury information, particularly among athletes with longer athletic experience. Second, injuries were not verified using medical records or clinical examinations, which may have affected the accuracy of injury classification and reporting. In addition, exposure-adjusted injury incidence rates could not be calculated because detailed training and competition exposure durations and athlete-exposure hours were unavailable. Actual exposure may have differed between wrestlers and judokas; therefore, the observed differences should not be interpreted as differences in injury risk. The findings represent retrospective self-reported injury frequencies rather than exposure-adjusted injury incidence rates. In addition, athletes with longer athletic careers may naturally report greater cumulative injury histories because of prolonged exposure duration, which should be considered when interpreting associations with multiple self-reported injury episodes. Furthermore, because athletes were able to report multiple injury episodes, some analyses were conducted at the injury-episode level. Although these analyses accounted for within-athlete clustering, residual dependence and unmeasured athlete-level heterogeneity may still have influenced the findings. Finally, the cross-sectional design limits causal interpretation regarding the relationships between training-related variables and multiple self-reported injury episodes.

Despite these limitations, the present study provides important comparative evidence regarding sport-specific injury characteristics among elite Turkish wrestlers and judokas competing at the national level. The findings may contribute to a better understanding of injury patterns and training-related factors associated with injury reporting in elite combat sports. In addition, the results may help support the development of targeted injury surveillance systems and the monitoring of training frequency and injury history among elite combat sport athletes.

## 6 Conclusion

The present study identified limited sport-related differences in self-reported injury-location patterns among elite wrestlers and judokas. After cluster adjustment and false discovery rate correction, statistically significant differences remained only for neck injuries and other shoulder injuries, both of which were reported less frequently by judokas than wrestlers. Athletic experience and weekly training frequency were significantly associated with reporting multiple injury episodes, which may reflect greater cumulative exposure across competitive careers. No statistically significant differences were detected between training and competition settings for the examined injury and health-problem categories. The findings emphasize the value of monitoring training frequency, cumulative exposure, and injury history in elite combat sport athletes. These results should be interpreted as self-reported injury frequencies and associations rather than exposure-adjusted injury incidence rates. Future prospective studies using standardized injury surveillance and detailed exposure measures are needed to clarify injury patterns in wrestling and judo.

## Data Availability

The raw data supporting the conclusions of this article will be made available by the authors, without undue reservation.
